# Alterations in Neuroinflammation, Microglia and Neuroplasticity in the Rat Hippocampus in a Combined Model of Periodontitis and Depression

**DOI:** 10.1111/cns.70669

**Published:** 2025-12-01

**Authors:** Javier Robledo‐Montaña, David Martín‐Hernández, Javier Cuenca‐Ortega, María Martínez, Leire Virto, Nagore Ambrosio, Eduardo Montero, María José Marín, David Herrera, Mariano Sanz, Juan C. Leza, Elena Figuero, Borja García‐Bueno

**Affiliations:** ^1^ Department of Pharmacology and Toxicology, Faculty of Medicine Complutense University of Madrid (UCM), Hospital 12 de Octubre Research Institute (Imas12), Neurochemistry Research Institute UCM (IUIN), and REIS (Network of Stress Research) Madrid Spain; ^2^ Biomedical Network Research Center of Mental Health (CIBERSAM) Institute of Health Carlos III Madrid Spain; ^3^ ETEP (Etiology and Therapy of Periodontal and Peri‐Implant Diseases) Research Group UCM Madrid Spain; ^4^ Department of Dental Clinical Specialties, Faculty of Dentistry UCM Madrid Spain; ^5^ Department of Anatomy and Embryology, Faculty of Optics and Optometry UCM Madrid Spain

**Keywords:** depression, hippocampus, microglia, neuroinflammation, periodontitis, synaptic plasticity

## Abstract

**Aims:**

The exact causes of major depressive disorder (MDD) are still debated, but its connection with inflammatory diseases and stress is well established. Emerging evidence suggests a potential link between periodontitis (gum disease) and MDD.

**Methods:**

Periodontitis (P) was induced in rats through oral rinses with the pathogenic bacteria *Porphyromonas gingivalis* and *Fusobacterium nucleatum* for 12 weeks, followed by 3 weeks of chronic mild stress (CMS) to induce depressive‐like behavior. Four experimental groups were established: periodontitis with CMS (*P* + CMS+), periodontitis without CMS (*P* + CMS‐), CMS without periodontitis (P‐CMS+), and control (P‐CMS‐). Inflammatory and synaptic plasticity‐related mediators were quantified in hippocampal samples. The number, morphology, and inflammatory phenotype of microglia were also evaluated by ultrastructural and fractal analyses.

**Results:**

*P* + CMS+ animals compared with controls showed: (1) increased protein expression of TLR‐4, phospho(p)‐nuclear factor kappa B (p‐NFκB)/NFκB ratio, and inducible nitric oxide synthase (iNOS); (2) decreased microglial number, shorter branch length, reduced complexity, and increased expression of iNOS; (3) decreased protein levels of BDNF and synaptophysin, and lower ratios of p‐protein kinase B (p‐Akt)/Akt and p‐mammalian target of rapamycin (p‐mTOR)/mTOR.

**Conclusion:**

Alterations in neuroinflammation and neuroplasticity in the hippocampus may contribute to the comorbidity between periodontitis and MDD, warranting further investigation.

## Graphical abstract

Cascade of effects within the HP of rats exposed to a combined model of periodontitis and depression. Akt, Protein kinase B; BDNF, Brain‐derived neurotrophic factor; CA1, cornu ammonis 1; CA3, cornu ammonis 3; DG, dentate gyrus; iNOS, Inducible nitric oxide synthase; LPS, lipopolysaccharide; mTOR, Mammalian target of rapamycin; NFκB, Nuclear factor kappa B; TLR‐4, toll‐like receptor 4; TrkB, Tropomyosin receptor kinase B.

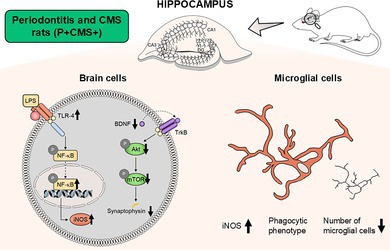

**Abbreviations**
Akt: protein kinase B
BDNF: brain‐derived neurotrophic factor
BSA: bovine serum albumin
CA1: *Cornu Ammonis* area 1
CA3: *Cornu Ammonis* area 3
CMS: chronic mild stress
CNS: central nervous system
DG: dentate gyrus
FC: frontal cortex
F‐IHC: fluorescence immunohistochemistry
Iba‐1: ionized calcium‐binding adapter molecule 1
iNOS: inducible nitric oxide synthase
KPBS: potassium phosphate‐buffered saline
LPS: lipopolysaccharide
MAPKs: mitogen‐activated protein kinases
MDD: major depressive disorder
mTOR: mammalian target of rapamycin
MyD88: myeloid differentiation primary response 88 (MYD88)
NFκB: nuclear factor kappa B
P: periodontitis
PBS: phosphate‐buffered saline
PFA: paraformaldehyde
PI3K: phosphatidylinositol‐3‐kinase
SEM: standard error of the mean
TLR‐4: toll‐like receptor‐4
TrkB: tropomyosin receptor kinase B

## 1 Introduction

Major depressive disorder (MDD) is a serious neuropsychiatric pathology projected to become the leading cause of disease burden worldwide by 2030, according to the World Health Organization [1]. Diagnostic criteria encompass the presence of a myriad of symptoms [2].

The etiology of MDD is considered multifactorial [3], with chronic and uncontrollable stress exposure as one of the main risk factors [4]. Several nonexclusive pathophysiological hypotheses have been proposed: (1) altered biogenic amine neurotransmission, (2) dysregulation of glutamate/gamma‐aminobutyric acid (GABA) receptors, (3) endocrine dysfunction, (4) neurotrophic factor alterations, (5) inflammation, and (6) impaired neuroplasticity and neurogenesis [5]. Probably, the incomplete understanding of the complex MDD pathophysiology contributes to the prevalence of treatment‐resistant depression, which affects at least 30% of those diagnosed [6] and emphasizes the urgent need for continued preclinical and clinical research in this field.

Among the brain structures implicated in the pathophysiology of MDD, hippocampal volume reduction is the most consistently replicated finding in neuroimaging studies [7]. The role of this limbic structure in MDD has garnered considerable attention [8] due to its critical involvement in mood regulation and cognition, as well as being suitable to explore putative alterations in stress‐related mechanisms, such as neuroinflammation and neuroplasticity [9, 10]. Both preclinical and clinical studies have demonstrated deficits in hippocampal neurogenesis, altered neuroplasticity, diminished cell survival, and increased neuroinflammation in MDD [11, 12]. Neuroinflammation has been proposed as a major regulator of neuroplasticity and is currently subject to pharmacological interventions with antidepressant drugs [13].

One of the unresolved questions about neuroinflammation in stress‐related neuropsychiatric pathologies is its origin [14]. Some studies suggest the idea that inflammatory processes are triggered by increased permeability of the gastrointestinal barrier, leading to bacterial translocation from the gut microbiome to other organs. This “*leaky gut*” has been directly involved in the pathophysiology of MDD and is now considered a promising therapeutic target [15].

In the last years, the gut microbiome concept has been expanded to the broader host microbiome notion, as other parts of the body are colonized by distinct ecosystems of microorganisms [16]. Specifically, several research groups, including ours, have evaluated the role of the oral microbiome related to periodontitis in stress‐related neuropsychiatric diseases [17]. Periodontitis is a chronic inflammatory condition initiated by subgingival biofilm and leading to dysbiosis, with the potential to trigger neuroinflammation through both concomitant cellular and humoral pathways [18]. In this line, the concept of a “*leaky mouth/gum*” has emerged due to the widening of the intercellular spaces between the epithelial cells and the rupture of the junctional epithelium on the periodontal pocket in patients with periodontitis, facilitating the translocation of bacteria and inflammatory mediators into the systemic circulation and distant organs, including the central nervous system (CNS) [19].

A positive epidemiological association between depression and periodontitis has been found in a recent metareview [20]. Additionally, one cohort study reported a hazard ratio of 1.73 (95% confidence interval [1.58; 1.89]) for developing depression in patients with periodontitis [21].

The relationship between oral health and psychiatric disorders appears to be complex, potentially involving multiple levels and bidirectional interactions [17, 22]. Therefore, preclinical and clinical studies are needed to disentangle this connection. It is worth mentioning that recent studies with rodents exposed to experimental models of periodontitis have reported neuroinflammation, impaired neuroplasticity, and reduced neurogenesis in the hippocampus (a relevant brain area involved in MDD pathophysiology), which have been directly related to cognitive deficits and depressive‐like behaviors [23, 24]. In this line, we have recently conducted a set of studies, validating the induction of periodontitis and signs of depressive‐like behavior, and demonstrating the presence of the periodontal pathogenic bacterial species *Fusobacterium* spp., signs of neuroinflammation, alterations in microglia and the endocannabinoid system, changes in synaptic plasticity‐related proteins, and modulation of key mediators involved in blood–brain barrier (BBB) permeability and sphingosine‐1‐phosphate (S1P) signaling in the frontal cortex (FC) of rats exposed to a combined model of periodontitis and depression [25, 26, 27].

Considering all this background, the present study aims to analyze whether the previously described combined model of periodontitis and depression produced also alterations in neuroinflammation‐related mechanisms in the hippocampus (HP), with a particular focus on changes in the number, morphology, and phenotype of microglia in the three main regions of the HP.

## 2 Methods

### 2.1 Animals

This study was designed according to the modified ARRIVE guidelines for preclinical in vivo research [28] and complied with Spanish and European Union regulations (European Communities Council Directive 86/609/EEC). The in vivo experimental part of the study was conducted at the Experimental Animal Center of the Complutense University of Madrid, following protocol approval by the regional authorities (PROEX 087/18) and the Ethical Committee of Animal Experimentation.

Male Wistar Hannover rats (HsdRccHan:Wist, from Envigo, Spain), weighing 230–280 g (9‐week‐old approx.), were maintained at a constant temperature of 24°C ± 2°C and relative humidity of 70% ± 5% in a 12‐h light–dark cycle (lights on at 8:00 AM), with free access to fresh tap water and standard pellet chow (A04 SAFE; Scientific Animal Food and Engineering, Augy, France) during all the experimental procedures. Animals were acclimatized to handling daily for 7 days prior to the start of the experiments.

### 2.2 Experimental Protocol

Four experimental groups resulted from the different combinations of periodontitis (P) and CMS: (a) control group (P‐CMS‐), (b) periodontitis group (*P* + CMS‐), (c) CMS group (P‐CMS+), and (d) periodontitis and CMS group (*P* + CMS+).

**FIGURE 1:**
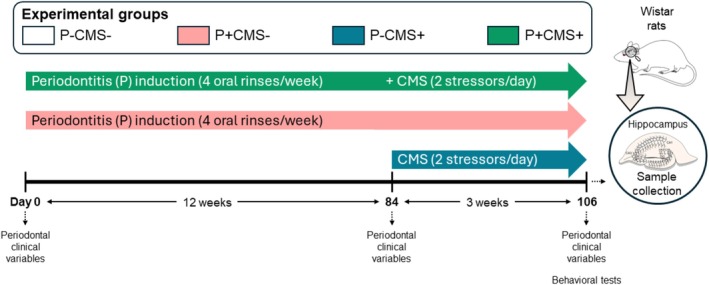
Experimental protocol. Schematic representation of the combined model of periodontitis and depression in rats. Periodontitis was induced via oral rinses with a solution of *Porphyromonas gingivalis* and *Fusobacterium nucleatum* for 12 weeks, followed by exposure to a chronic mild stress (CMS) model for 3 weeks.

Periodontitis was induced first (12 weeks) using an oral rinse method, and animals were subsequently exposed to CMS (3 weeks). During the CMS, oral rinses were continued (Figure 1).

Both in vivo protocols have been previously reported and validated individually [29, 30] and in combination [25, 26], including the periodontal outcomes and behavioral results of the experimental groups used in the present study [25]. The experimental periodontitis model consisted of inoculating two known periodontal pathogens, *P. gingivalis* ATCC W83K1 and *F. nucleatum* DMSZ 20482, through oral rinses. The CMS consisted of a series of different stressors such as food deprivation, water deprivation, cage tilting, soiled cage, grouped housing after a period of water deprivation, stroboscopic illumination [150 flashes/min], and intermittent illumination every 2 h, changed daily (two stressors/day) and given unpredictably over 21 days, plus one extra day to maintain the stress exposure during the behavioral tests.

Forty‐eight male rats were randomly assigned to the four groups (*n* = 12 rats/group). Two rats died after anesthesia administration at baseline (P‐CMS‐ group) and two after periodontitis induction (*P* + CMS‐ and P‐CMS+ groups). Therefore, 44 rats completed the in vivo experimental phase. Of these, 12 were used for fluorescence immunohistochemistry (F‐IHC) (*n* = 3 rats/group) and 30 for western blot assays (*n* = 7–8 rats/group). Two animals (1 from the P‐CMS‐ group and 1 from the *P* + CMS‐ group) were excluded from the initial cohort of 32 for methodological pitfalls (see next section, *Tissue specimens*).

### 2.3 Tissue Specimens

Samples were collected after terminal anesthesia, at the end of the final stress session, using sodium pentobarbital (320 mg/kg i.p.; Vetoquinol, Madrid, Spain) between 2:00 and 3:00 PM to minimize circadian rhythm effects. Animals were divided into two sets, one for F‐IHC and the other for biochemical analyses.

For F‐IHC, rats were perfused via the ascending aorta with 200 mL of saline solution followed by 200 mL of 4% paraformaldehyde (PFA) in 0.1 M phosphate‐buffered saline (PBS) (pH = 7.4). Brains were collected, postfixed in 4% PFA overnight at 4°C, cryoprotected with 30% sucrose, and frozen. Coronal sections of 30 μm from the FC were prepared using a microtome and stored at −40°C immersed in a cryopreservation solution.

For biochemical analyses, blood was collected via cardiac puncture, anticoagulated with ethylenediaminetetraacetic acid (EDTA, 1% w:v, 1 vol EDTA per 50 vol blood) and centrifuged at 366 g for 15 min at room temperature to isolate plasma, which was stored at −80°C. Brains were harvested after decapitation, and the left hippocampus was dissected and immediately frozen at −80°C. Total homogenates were prepared by homogenizing hippocampal tissue in PBS 1× (pH = 7) with a protease inhibitor cocktail (Complete Roche, Basel, Switzerland) using the TissueLyser LT (QIAGEN, Hilden, Germany) at 50 Hz for 4 min, and centrifuged at 19,083 *g* for 10 min. The supernatant was the hippocampal protein extract used for western blot analysis.

### 2.4 Fluorescence Immunohistochemistry (F‐IHC)

Three hippocampal regions—CA1, CA3, and DG—were analyzed in one brain slice per animal. Antigen retrieval was performed by incubating sections in a sodium citrate solution (pH = 6.0) for 40 min, ranging from 40°C to 65°C. Sections were washed with 0.02 M potassium phosphate‐buffered saline (KPBS), immersed in 0.1 M glycine for 20 min to reduce autofluorescence, washed again, blocked with 10% bovine serum albumin (BSA) in KPBS with 0.1% Triton X‐100 for 60 min, incubated overnight with primary antibody in 10% BSA KPBS, washed again, incubated with secondary antibody in 10% BSA KPBS, and mounted with Fluoroshield containing 40,6‐diamidino‐2‐phenylindole dihydrochloride (DAPI). Antibody titration was optimized to prevent nonspecific interactions and establish the best immunosignal. Negative controls for each antibody ensured the absence of nonspecific fluorescent signals. Two 20× confocal images per region were obtained in the confocal microscope TCS SP8 (Leica Microsystems, Wetzlar, Germany) at CAI‐UCM Flow Cytometry and Fluorescence Microscopy Unit.

#### 2.4.1 Microglial Morphological Analysis

Microglia were labeled with an anti‐ionized calcium‐binding adapter molecule 1 (Iba‐1) (ab108539, Abcam, dilution 1:1000) and a secondary Alexa fluor 555‐conjugated donkey anti‐rabbit antibody (A31572; Life Technologies, 1:1000). A total of 90 microglial cells per group were evaluated (5 cells/microphotography × 2 microphotography/section × 3 section/rat × 3 rat/group). Microglial count (cells/region) was calculated as the average of microglial cells across the CA1, CA3, and DG regions. Morphological analysis was based on the protocol by Vargas‐Caraveo et al. [31] using the Fiji Image J software (NIH, Bethesda, MD, USA). Binary images were created by threshold adjustment, and single‐cell images were isolated for fractal and skeleton analysis using the FracLac and Analyze Skeleton plugins, respectively. Parameters such as the number and length of branches, cellular area, lacunarity, fractal dimension, and density were evaluated [27].

#### 2.4.2 Inducible Nitric Oxide Synthase (iNOS) Microglial Expression

For the analysis of iNOS microglial expression, primary antibodies were anti‐iNOS (BD610329 BDBioscience, 1:1000) and anti‐Iba‐1. Respectively, secondary antibodies were Alexa Fluor 488 conjugated goat anti‐mouse (A11001; Life Technologies, 1:1000) and Alexa Fluor 555 conjugated donkey anti‐rabbit (A31572; Life Technologies). A total of 72 microphotographs were analyzed (two microphotography/region × three region/rat × three rat/group = 18 microphotography/group). The threshold was applied to gray images to identify the number of Iba‐1+ cells. iNOS fluorescence intensity was calculated as the ratio of the general mean value of the iNOS signal to the number of parenchymal microglial cells (Iba‐1+ cells) in each image.

### 2.5 Western Blot (WB)

Protein levels of the hippocampal homogenates were adjusted using the Bradford method based on the principle of protein–dye binding. After mixing them with Laemmli sample buffer (Bio‐Rad, Hercules, CA, USA), 11.25 μg of protein was loaded and size‐separated in 8% sodium dodecyl sulfate‐polyacrylamide gel electrophoresis (90 V). Gels were transferred to nitrocellulose membranes using the Trans‐Blot Turbo Transfer System (Bio‐Rad, Hercules, CA, USA). Membranes were blocked in Tris‐buffered saline containing 0.1% Tween 20 and 5% BSA for 1 h and incubated overnight at 4°C with specific primary antibodies against Toll‐like receptor‐4 (TLR‐4) (SPC‐200D StressMarq, 1:750, 2.5% BSA), phospho(p)‐nuclear factor kappa B (p‐NFκB) (MA5‐15160 ThermoFisher Scientific, 1:750, 2.5% BSA), NFκB (6956 Cell Signaling, 1:750, 2.5% BSA), iNOS (ab15323 Abcam, 1:750, 2.5% BSA), brain‐derived neurotrophic factor (BDNF) (ab108319 Abcam, 1:1000, 2.5% BSA), tropomyosin receptor kinase B (TrkB) (ab18987 Abcam, 1:1000, BSA 5%), PI3K (sc7189 Santa Cruz, 1:1000, 5% BSA), p‐protein kinase B (p‐Akt) (4060 Cell Signaling, 1:1000, 2.5% BSA), Akt (4691 Cell Signaling, 1:2000, 2.5% BSA), p‐mammalian target of rapamycin (p‐mTOR) (phosphorylation site in residue SER 2448) (5536 Cell Signaling, 1:1000, 5% BSA), mTOR (2983 Cell Signaling, 1:1000, 5% BSA), synaptophysin (S5768 Sigma Aldrich, 1:2000), and postsynaptic density protein 95 (PSD95) (sc32290 Santa Cruz, 1:750, 2.5 BSA). After washing, membranes were incubated with the respective horseradish peroxidase‐conjugated secondary antibodies (anti‐rabbit IgG‐HRP sc2357 Santa Cruz, 1:2000; anti‐mouse‐HRP sc516102 Santa Cruz, 1:2000, 2.5% BSA) for 90 min at room temperature. Finally, the membranes were developed with the ECL Prime kit following the manufacturer's instructions (Cytiva Marlborough, MA, USA). Blots were imaged in a ChemiDoc (BioRad, Hercules, CA, EEUU) and quantified by densitometry using the Fiji Image J package. All densitometry data were obtained in arbitrary units of optical density and expressed as a percentage of the control group (100%). Several exposure times were analyzed to ensure the linearity of the band intensities. The loading controls used were beta‐Actin (A5441 Sigma, 1:10000).

### 2.6 Plasma Cytokines

Interleukin (IL)‐1β, Tumor necrosis factor‐alpha (TNF‐α), IL‐6, interferon‐gamma (IFN‐γ) and IL‐10, and monocyte chemoattractant protein 1 (MCP‐1) were measured by means of high‐sensitivity multiplex map rat immunoassays (Merck) using a flow cytometry analyzer (Luminex‐200 System; Luminex Corporation, Oosterhout, The Netherlands). Results were measured using xPonent software (Luminex corporation) and were expressed as picograms per milliliter (pg/mL).

### 2.7 Statistical Analysis

Information about the sample size calculation was reported by Martínez et al. [25]. Briefly, 48 animals (12 animals per group) were considered necessary to detect a difference of 1.6× sigma in the expression of neuroinflammatory mediators at the protein level, with a standard deviation (SD) of 25. Only 12 animals (3 animals per group) were considered for the immunofluorescence studies.

Data were expressed as mean ± standard error of the mean (SEM). The robust regression and outlier removal (ROUT) method (Q = 1%) identified significant outliers. The normality of the distribution was evaluated with the Shapiro–Wilk test, and the Brown–Forsythe test checked variance homogeneity. If data fitted a Gaussian distribution, a one‐way ANOVA followed by a Tukey post hoc test was used when equal variances could be assumed, while a Brown–Forsythe ANOVA test followed by Tamhane's T2 test was run in cases where variances were not homogeneous. If a Gaussian distribution could not be assumed, a nonparametric Kruskal–Wallis test with Dunn's test with adjustments for multiple comparisons was used. A *p* value ≤ 0.05 was considered to be statistically significant. Data were analyzed using GraphPad Prism 9 (GraphPad Software, San Diego, CA, USA).

## 3 Results

### 3.1 Neuroinflammation. Characterization of the TLR‐4 Signaling Pathway

**FIGURE 2:**
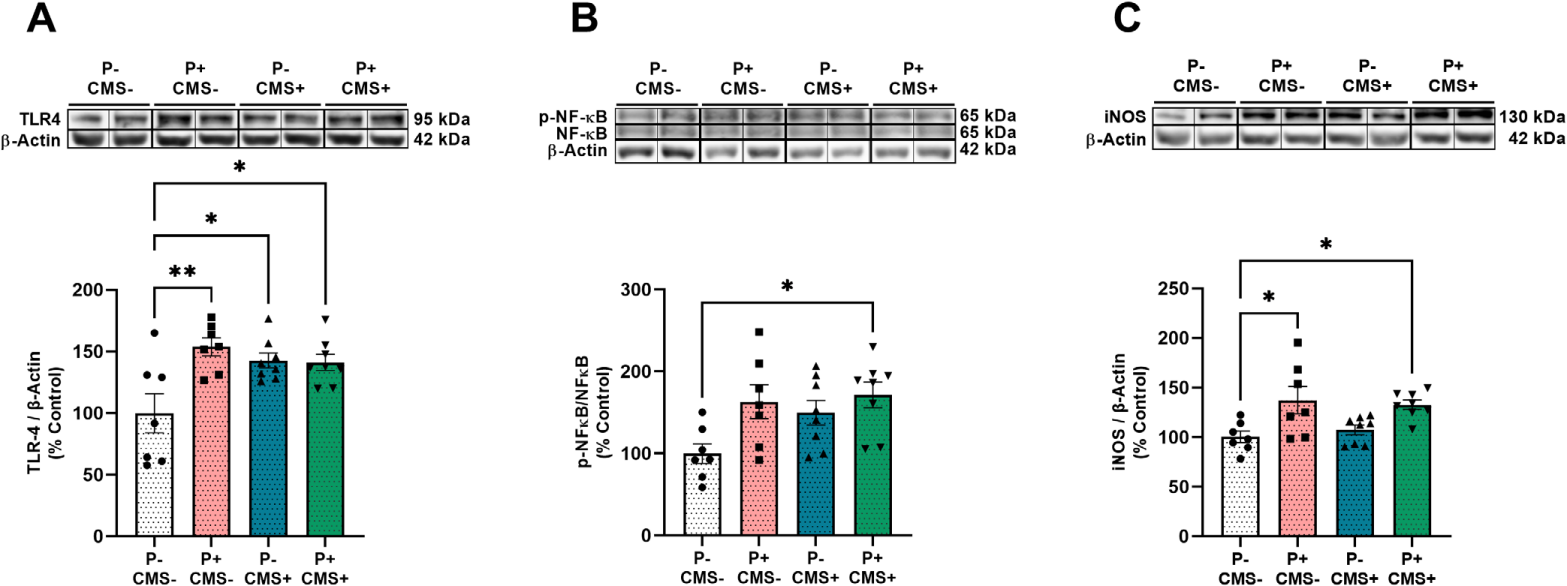
Neuroinflammatory markers in the hippocampus of rats in control conditions (P‐CMS‐), and after periodontitis induction (*P*+CMS‐), chronic mild stress exposure (P‐CMS+), and both protocols combined (*P*+CMS+). Protein expression of Toll‐like receptor‐4 (TLR‐4) (A), phospho‐nuclear factor kappa B (p‐NFκB)/NFκB ratio (B), inducible nitric oxide synthase (iNOS) (C), in hippocampal samples by western blot (WB). For TLR‐4 and iNOS, densitometric data of the band of interest were normalized to β‐Actin and expressed as a percentage of control. *p‐NFκB/NFκB* values were normalized to the loading control (*β‐Actin*), respectively. Then we looked at the phospho/total ratio using the normalized values. Data are finally expressed as a percentage of control. Data are expressed as mean ± standard error of the mean (SEM) of 7–8 rats per group. *p < 0.05, **p < 0.01. One‐way ANOVA with Tukey post hoc for TLR‐4, p‐NFκB/NFκB, and iNOS. Blots were cropped (black lines) to enhance the clarity and conciseness of the presentation. Original blot images can be found in Supporting Information.

One of the most relevant pro‐inflammatory pathways activated in the CNS during stress‐related neuropathologies is orchestrated by the TLR‐4 innate immune receptor. In our experimental conditions, western blot analysis revealed that the protein expression of TLR‐4 in hippocampal samples was lower in the P‐CMS‐ group, compared with the other groups (Figure 2A). The most described TLR signaling pathway is myeloid differentiation primary response 88 (MyD88)–dependent, that regulates the activation of the main pro‐inflammatory mediator NFκB. The ratio p‐NFκB/NFκB was higher in the hippocampus of the *P* + CMS+ group, compared with the P‐CMS‐ control group (Figure 2B). A direct consequence of NFκB activation is the upregulation of the pro‐inflammatory enzyme iNOS, whose protein expression was increased in both the *P* + CMS‐ and P + CMS+ groups, compared with P‐CMS‐ (Figure 2C).

### 3.2 Quantification and Morphological Analysis of Microglia

**FIGURE 3:**
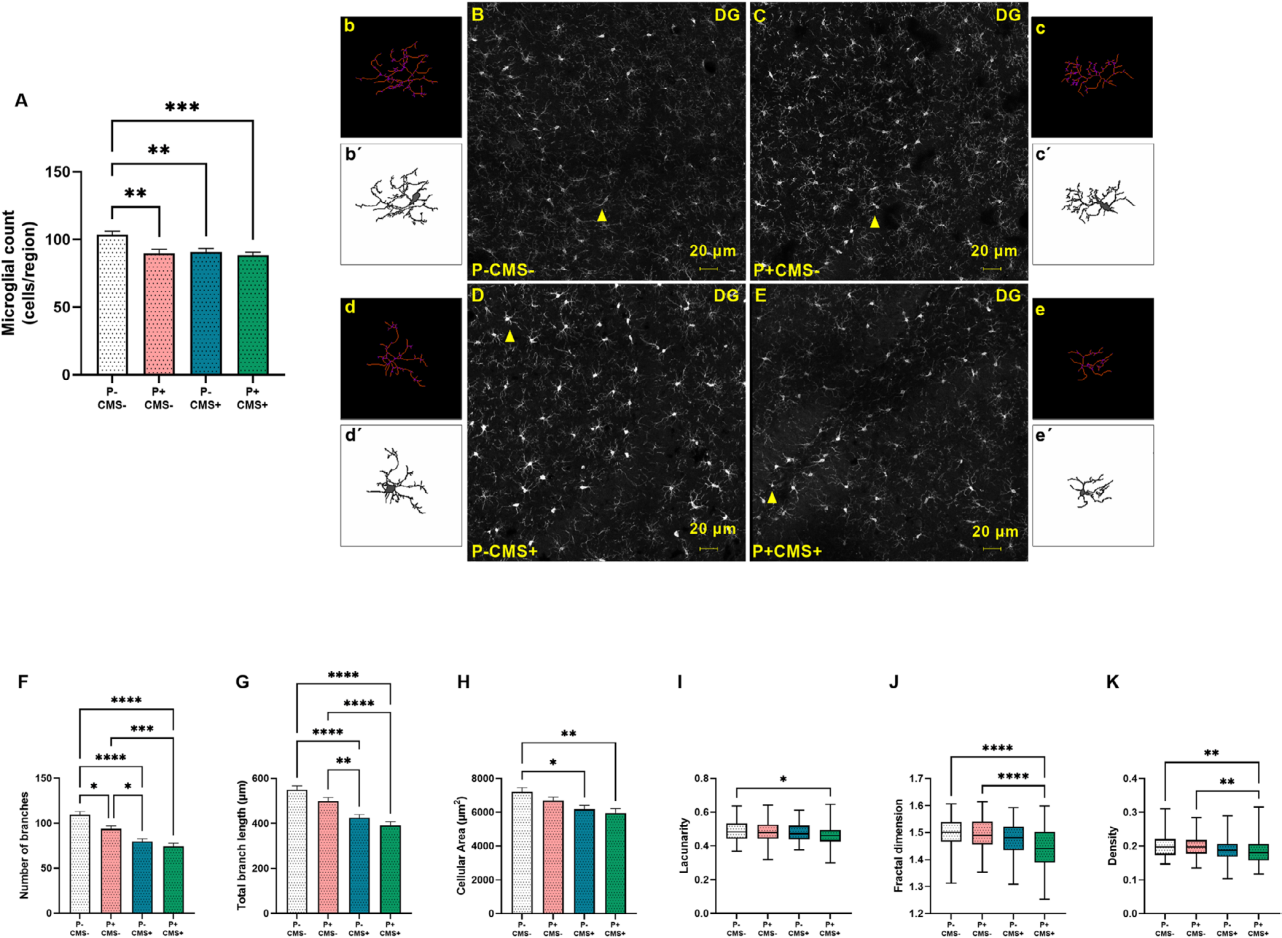
Analysis of global microglial count and morphology in the dentate gyrus (DG) region of the hippocampus of rats in control conditions (P‐CMS‐), and after periodontitis induction (*P* + CMS‐), chronic mild stress exposure (P‐CMS+), and both protocols combined (*P* + CMS+). Immunofluorescence of ionized calcium‐binding adapter molecule 1 (Iba‐1) in representative images of 30 μm‐thick sections of rat brain hippocampus. Global quantification of Iba‐1+ parenchymal microglial cells (A). Six microphotographs per animal (3 animals per group) were analyzed at 20X magnification: P‐CMS‐ (B), *P* + CMS‐ (C), P‐CMS+ (D), and *P* + CMS+ (E). Skeletonized and binarized outlined images of microglia in the DG (b–e, b'–e'). Yellow arrowheads indicate representative cells. Statistical analysis of the number of microglial branches (F), total branch length (G), cell area (H), lacunarity (I), fractal dimension (J) and density (K). Data are presented as mean ± standard error of the mean (SEM) of 90 microglia per group. *p < 0.05; **p < 0.01; ***p < 0.001; ****p < 0.0001. One‐way ANOVA with Tukey post hoc test for lacunarity, and Kruskal–Wallis test with Dunn's multiple comparisons for the remaining parameters. Scale bars = 20 μm.

First, we quantified the number of microglial cells in the CA1, CA3, and DG regions of the hippocampus. Considering the sum of microglial counts across all three hippocampal regions, revealed that the microglial cell number decreased in all experimental groups, compared with the control animals (P‐CMS‐) (Figure 3A).

Morphological characteristics of microglia (Figure 3B–E) were examined through skeletal and fractal analyses using skeletonized (Figure 3b–e) and outlined (Figure 3b'–e') representations in the DG. Skeletal analysis found alterations in the number and length of microglial branches (Figure 3F,G). Specifically, there was a notable reduction in the number of microglial branches in all experimental groups, compared with the control group, with the *P* + CMS+ group showing the most pronounced decrease (Figure 3F). A similar pattern was observed for the total branch length, though no statistically significant differences were detected between the P‐CMS‐ and *P* + CMS‐ groups (Figure 3G).

Several additional parameters (cellular area, lacunarity, fractal dimension, and density) were assessed to further characterize microglial morphology in the DG (Figure 3H–K). The cellular area was reduced in both P‐CMS+ and *P* + CMS+ groups, compared with the control group, being the *P* + CMS + group the one exhibiting the greatest decrease (Figure 3H).

Lacunarity, which reflects morphological heterogeneity, was significantly lower in the *P* + CMS + group, compared with the control (Figure 3I). Fractal dimension and density, defined as the foreground/hull area ratio, assessed the complexity of microglial morphology (Figure 3J,K). The fractal dimension revealed reduced complexity in the *P* + CMS + group, compared with the control (Figure 3J). The density followed the same pattern, with reduced values in the *P* + CMS+ group, suggesting a reorganization of the branches into a retracted state (Figure 3K). Both fractal dimension and density emphasized the diminished complexity of microglia in the *P* + CMS + group, compared with the other groups.

### 3.3 Inflammatory Phenotypical Analysis of Microglia: iNOS Expression

**FIGURE 4:**
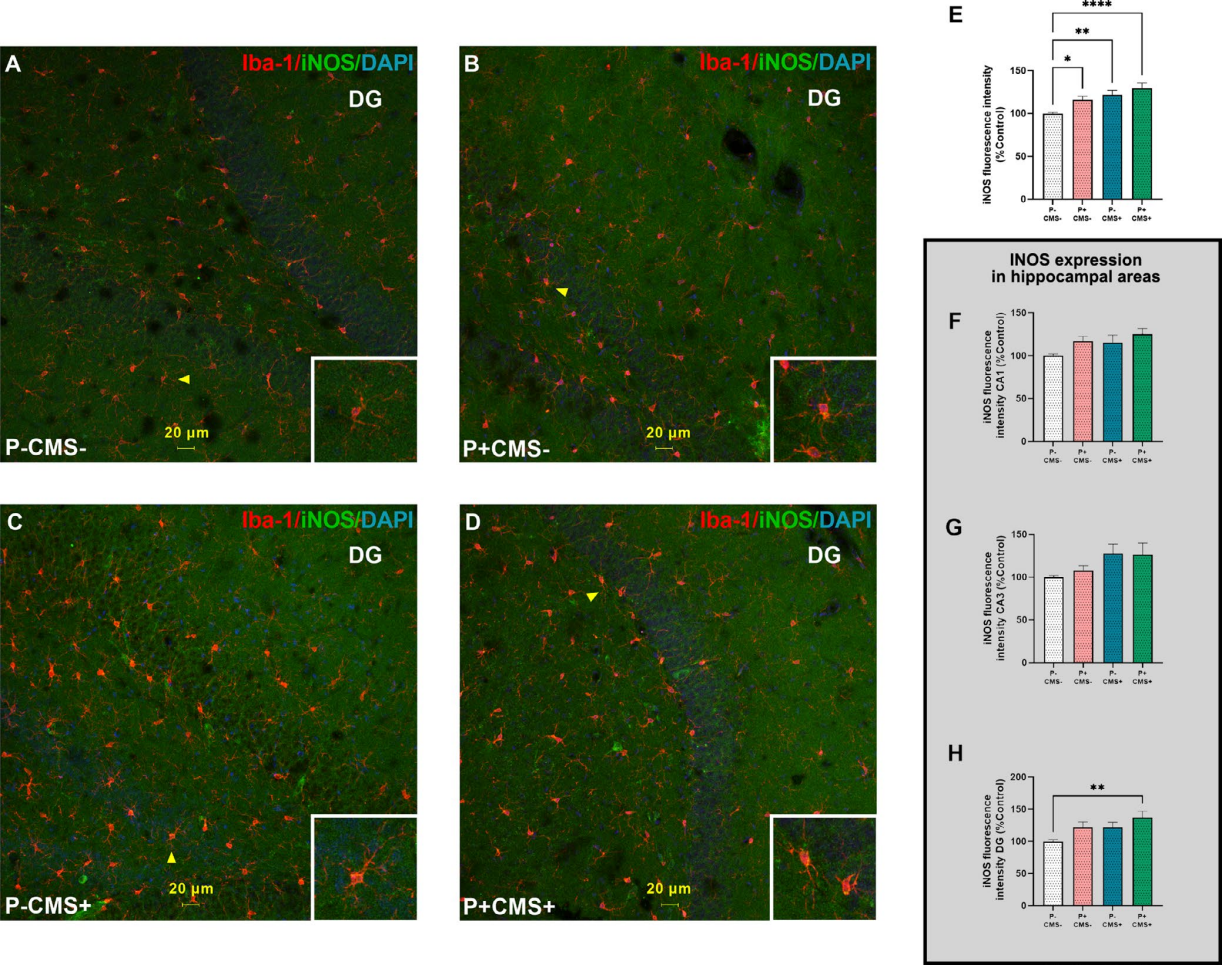
Inducible nitric oxide synthase (iNOS) immunoreactivity in parenchymal microglia of the hippocampus of rats in control conditions (P‐CMS‐), and after periodontitis induction (*P* + CMS‐), chronic mild stress exposure (P‐CMS+), and both protocols combined (*P* + CMS+). Immunofluorescence of ionized calcium‐binding adapter molecule 1 (Iba‐1) (red), iNOS (green), and 4′,6‐diamidino‐2‐phenylindole dihydrochloride (DAPI) in nuclei (blue) was performed in representative images of 30 μm‐thick sections of rat brain hippocampus from P‐CMS‐ (A), *P* + CMS‐ (B), P‐CMS+ (C), and *P* + CMS+ (D). A‐D images are from dentate gyrus (DG) region. Yellow arrowheads indicate representative cells. Quantitative analysis of iNOS expression in the Iba‐1 + cells of all hippocampal regions (E). iNOS immunoreactivity in *Cornu Ammonis* area 1 (CA1) (F), *Cornu Ammonis* area 1 (CA3) (G) and DG (H) regions. Data are presented as mean ± standard error of the mean (SEM) of 18 microphotographs per group (E) and 6 microphotographs per group (F–H). *p < 0.05; **p < 0.01; ****p < 0.0001. One‐way ANOVA with Tukey post hoc test for iNOS immunoreactivity in CA1, and Kruskal–Wallis test with Dunn's multiple comparisons for the remaining parameters. Scale bars = 20 μm.

Building on the previous characterization of parenchymal microglial morphology, we aimed to corroborate the pro‐inflammatory phenotype of microglia in the HP across our experimental groups. To this end, we investigated the microglial expression of iNOS (a widely known pro‐inflammatory cellular marker) in the CA1, CA3, and DG regions of the hippocampus (Figure 4A–D). When iNOS immunoreactivity was analyzed collectively across the three regions, a significant increase was observed in all experimental groups, compared with the control group (P‐CMS‐) (Figure 4E). However, when the analysis was stratified by hippocampal regions (Figure 4F–H), a significant increase in iNOS immunosignal in microglia was found only in the *P* + CMS+ groups, compared with control animals, reaching statistical significance only in the DG (Figure 4H).

### 3.4 Neuroplasticity. Characterization of the BDNF Signaling Pathway

The role of BDNF in neuroplasticity, particularly in MDD and other stress‐related neuropsychiatric diseases, has been extensively explored [32]. Our final objective was to evaluate whether our experimental conditions, either alone or in combination, altered the expression of elements in the BDNF–TrkB–PI3K–Akt–mTOR signaling pathway in the hippocampus.

**FIGURE 5:**
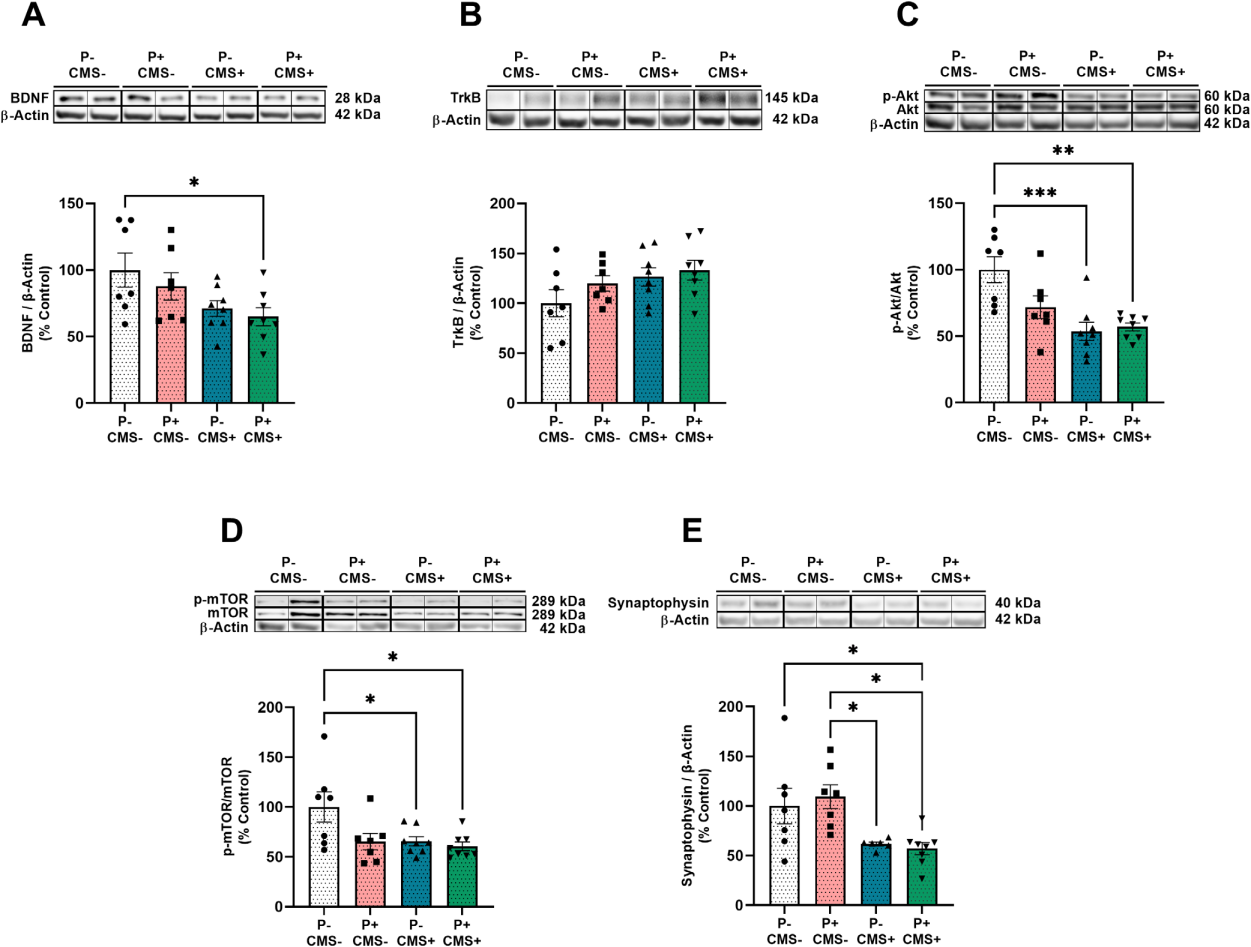
Neuroplasticity markers in the hippocampus of rats in control conditions (P‐CMS‐), and after periodontitis induction (*P* + CMS‐), chronic mild stress exposure (P‐CMS+), and both protocols combined (*P* + CMS+). Protein expression of brain‐derived neurotrophic factor (BDNF) (A), tropomyosin receptor kinase B (TrkB) (B), phosphoprotein kinase B (p‐Akt)/Akt ratio (C), p‐mammalian target of rapamycin (p‐mTOR)/mTOR ratio (D) and synaptophysin (E) in hippocampal samples by western blot (WB). Densitometric data of the band of interest were normalized to β‐Actin and expressed as a percentage of control for BDNF, TrkB and synaptophysin. Akt/mTOR and total AKT/mTOR values were normalized to the loading control (*β‐Actin*), respectively. Then, we looked at the phospho/total ratio using the normalized values. Data are finally expressed as a percentage of control. Data are mean ± standard error of the mean (SEM) of 7–8 rats per group. *p < 0.05, **p < 0.01, ***p < 0.001. One way‐ANOVA with Tukey post hoc. Blots were cropped (black lines) to improve the clarity and conciseness of the presentation. Original blot images can be found in Supporting Information.

BDNF protein levels diminished in the *P* + CMS+ group, compared with P‐CMS‐ (Figure 5A). No statistically significant differences were found in TrkB receptor (Figure 5B) or PI3K (data not shown) expression among groups. The p‐Akt/Akt and p‐mTOR/mTOR ratios were lower in groups subjected to CMS, with or without previous periodontitis, compared with the control group (P‐CMS‐) (Figure 5C,D).

The BDNF/TrkB mediated PI3K/Akt/mTOR signaling pathway has been recently implicated in neuroplasticity through the upregulation of synaptic proteins, such as synaptophysin and PSD95 [33]. Under our experimental conditions, the *P* + CMS+ group exhibited lower synaptophysin levels than P‐CMS‐ and *P* + CMS‐, while the P‐CMS+ group showed reduced levels only compared with *P* + CMS‐ (Figure 5E). No statistically significant differences in PSD95 protein expression were observed among groups (data not shown).

## 4 Discussion

Growing evidence highlights comorbidity between periodontitis and systemic inflammatory‐related conditions, such as cardiovascular and metabolic diseases [34]. In recent years, particular attention has been paid to its association with neurodegenerative, and neuropsychiatric disorders, especially Alzheimer's disease [35]. Researchers are now focusing on the causal directionality between periodontitis and mental disorders, such as MDD, through complementary approaches. Some studies suggest that periodontitis may be a risk indicator for MDD via microbiological or inflammatory mechanisms [36], while others explore how MDD negatively impacts oral health [37]. A holistic, bidirectional perspective has also been proposed [38].

Our previous studies have demonstrated alterations in neuroinflammation, such as TLR‐4 and S1P pathways, in the FC of rats exposed to a combined protocol of periodontitis and CMS [25, 26, 27]. Expanding on those findings using the same set of animals, we now report similar effects on TLR‐4 and BDNF signaling in the hippocampus [9].

The pro‐inflammatory TLR‐4/NFκB/iNOS signaling is overactivated in this region under our experimental conditions. Both periodontitis and CMS can upregulate this pathway in the hippocampus even separately [39, 40].

Additionally, the classic M1 pro‐inflammatory mediator iNOS was upregulated in parenchymal microglia both in FC and hippocampus [41]. However, the effects on microglial number and morphology were region‐specific [26, 27], underscoring the growing interest in microglial regional heterogeneity [42]. While resting microglia in the FC and hippocampus are highly ramified, hippocampal microglia appear more sensitive to bacterial infections than those in the hypothalamus or FC in aging rats [43]. In this model, hippocampal microglia exhibited a phagocytic morphology characterized by fewer and shorter processes emerging from rounder cell bodies. In contrast, FC microglia displayed a hyper‐ramified morphotype defined by more abundant, longer, and thicker processes arising from larger, lobular, and irregularly shaped cell bodies (for a direct comparison of the microglial morphology between both brain regions please see our previous study [27]). Both morphotypes align with the spectrum of activated microglia, consistent with prior studies on periodontitis and CMS in rodents [44]. Further comparative studies are needed to elucidate whether these differences in the number and morphology of microglial cells between FC and hippocampus reflect functional distinctions.

The observed reduction in the microglial count in the hippocampus warrants further discussion. The hippocampus typically has a higher microglial density than other brain regions, such as the cerebral cortex [45]. Although periodontitis models generally activate hippocampal microglia, few studies examine its impact on microglial density. Chronic systemic *P. gingivalis* administration has been shown to increase microglial number in the hippocampus [46], contrary to our findings. Differences in dosage, administration route, or treatment duration may explain these discrepancies. Chronic stress, however, has been identified as a major regulator of microglial dynamics [47]. While acute or subacute stress promotes microglial proliferation in the hippocampus and other stress‐sensitive brain areas [48], prolonged stress exposure reduces microglial numbers in certain regions, possibly due to delayed activation of proapoptotic pathways [44]. In this line, a 5‐week CMS protocol in C57BL/6J male mice reduced Iba‐1+ cells in the hippocampus, especially in the CA1 and DG subregions [49].

The inhibitory effects of our combined experimental model on the BDNF–TrkB‐PI3K‐Akt–mTOR pathway in the hippocampus mirror those observed in the FC [25, 27]. Previous studies have shown that different models of periodontitis disrupt BDNF signaling in the hippocampus, contributing to depressive‐like behaviors and cognitive deficits [46, 50]. However, in our study, animals exposed only to periodontitis (*P*+CMS‐) exhibited modest effects on the BDNF pathway, suggesting that CMS is the dominant factor driving the observed changes in the combined group (*P*+CMS+). In fact, the inhibitory impact of chronic stress on BDNF signaling in the hippocampus and its behavioral and cognitive consequences is well‐documented in MDD and other stress‐related neuropsychopathologies [51]. Furthermore, this pathway is a target for antidepressant treatment in animal models, with evidence especially strong for ketamine [52]. In the clinical area, there is debate regarding whether selective serotonin reuptake inhibitors depend on BDNF for their efficacy [53], and whether circulating BDNF is a reliable biomarker [54].

As observed previously in the FC [27], the combined model is the only experimental condition showing a reduction of synaptophysin protein levels (a marker of synaptic integrity) compared to control conditions in HP samples. The analysis of all the comparisons made between groups suggests that this inhibitory effect appears closely related to CMS exposure, leaving periodontitis induction as a modulating stimulus. In this line, other authors have shown that chronic stress reduced synaptophysin expression in the hippocampus, correlating with depressive‐like behaviors and cognitive deficits [55]. mTOR and Akt have been proposed as potential regulators of synaptophysin in the hippocampus [33, 56]. In this line, modulating PI3K‐Akt/mTOR signaling is in the spotlight for psychiatric treatments [57].

Nonetheless, certain limitations must be acknowledged. First, complementary methodologies are needed to identify the involvement of other cell types in neuroinflammation and neuroplasticity. As an illustrative example, new findings demonstrate that astrocytes play an active role in BDNF physiology [58]. Second, the descriptive nature of our study is insufficient to draw an unequivocally spatiotemporal cascade of events from neuroinflammation to neuroplasticity, necessitating further studies focusing on therapeutic targets (including a “periodontitis treatment group” or an “anti‐inflammatory intervention group”) to determine whether the observed changes are reversible or therapeutically relevant. In this line, Omics approaches are also needed to identify differential genes/proteins linking periodontitis and depression. Considering the dysregulation of the hypothalamic–pituitary–adrenal stress axis (reflected by the increase in plasma corticosterone and in brain glucocorticoid receptor levels), and the depressive‐like behavior previously found in the combined *P*+CMS+ group [25], further studies are needed to disentangle the systemic inflammation's complex role in the neuroinflammation observed in our experimental conditions (it is worth mentioning that no differences were found in the plasma levels of the pro‐inflammatory cytokines IL‐1β, TNF‐α, IL‐6 and IFN‐γ, and of the anti‐inflammatory cytokine IL‐10 between groups) (data not shown), and whether there is a direct correlation between molecular changes in HP and phenotypic outcomes.

Finally, sex differences and behavioral tests assessing cognitive function would have been valuable in evaluating the repercussions of the observed molecular changes.

## 5 Conclusion

The results from the present investigation highlight key molecular and cellular alterations in the hippocampus of animals exposed to periodontitis and CMS, including upregulation of the pro‐inflammatory TLR‐4 signaling pathway, reduced microglial density coupled with the induction of an activated phenotype, downregulation of the BDNF signaling pathway, and decreased levels of the synaptic plasticity‐associated protein synaptophysin (see graphical abstract). These findings identify promising candidates involved in neuroinflammation and neuroplasticity, offering insights into the intricate interplay between oral health and psychiatric disorders. Moreover, they may serve as alternative therapeutical targets to explore in the future.

## Author Contributions

**Javier Robledo‐Montaña:** conceptualization, methodology, validation, formal analysis, investigation, data curation, writing – review and editing, visualization. **David Martín‐Hernández:** conceptualization, methodology, validation, formal analysis, investigation, writing – original draft, visualization, supervision, project administration. **Javier Cuenca‐Ortega:** validation, formal analysis, investigation, data curation, writing – review and editing. **María Martínez:** investigation, writing – review and editing. Leire Virto: investigation, writing – review and editing. **Nagore Ambrosio:** investigation, writing – review and editing. **Eduardo Montero:** investigation, writing – review and editing. **María José Marín:** investigation, writing – review and editing. **David Herrera:** resources, writing – review and editing. **Mariano Sanz:** resources, writing – review and editing. **Juan C. Leza:** conceptualization, methodology, resources, writing – review and editing, supervision, project administration. **Elena Figuero:** conceptualization, methodology, resources, writing – review and editing, supervision, project administration. **Borja García‐Bueno:** conceptualization, methodology, validation, formal analysis, investigation, resources, writing – original draft, visualization, supervision, project administration, funding acquisition.

## Funding

This study was funded through a research grant from Santander–University Complutense of Madrid Projects in 2017 (PR41/17–20979; principal investigator: Elena Figuero), by MINECO‐FEDER Funds (PID2019‐109033RB‐100 and PID2022‐137932NB‐I00; principal investigators: Juan Carlos Leza and Elena Figuero), and CIBERSAM/ISCIII.

## Ethics Statement

This study was designed according to the modified ARRIVE guidelines for preclinical in vivo research [28] and following the Spanish and European Union regulations (European Communities Council Directive 86/609/EEC). The in vivo experimental part of the study was carried out in the Experimental Animal Center of the Complutense University of Madrid after its protocol was approved by the regional authorities (PROEX 087/18) and the Ethical Committee of Animal Experimentation.

## Consent

The authors have nothing to report.

## Conflicts of Interest

The authors declare no conflicts of interest.

## Supporting information


**Data S1:** cns70669‐sup‐0001‐DataS1.docx.

## Data Availability

Research data can be found in Docta Complutense, an Open Access Institutional Repository of the Complutense University of Madrid, with the following reference: https://hdl.handle.net/20.500.14352/120396.
