# Supplementary material for: Alterations in Neuroinflammation, Microglia and Neuroplasticity in the Rat Hippocampus in a Combined Model of Periodontitis and Depression
Source: CNS Neurosci Ther. 2025 Dec 1;31(12):e70669. doi: 10.1111/cns.70669 (PMC12668902; doi:10.1111/cns.70669)

Blots were imaged using a ChemiDoc™ system (BioRad®, Hercules, CA, USA) and quantified through densitometry with the Fiji Image J® package (NIH, Bethesda, MD, USA).

All densitometry readings were obtained in arbitrary units of optical density and expressed as a percentage of the control group (100%) and represented as the media of 2-4 different replicates.

Beta-actin (β-actin) (sc-47778, Santa Cruz Biotechnology; 1:5000) was utilized as loading control.

Representative blots are depicted in Figures 2 and 5 and the selected ones are highlighted with red below.


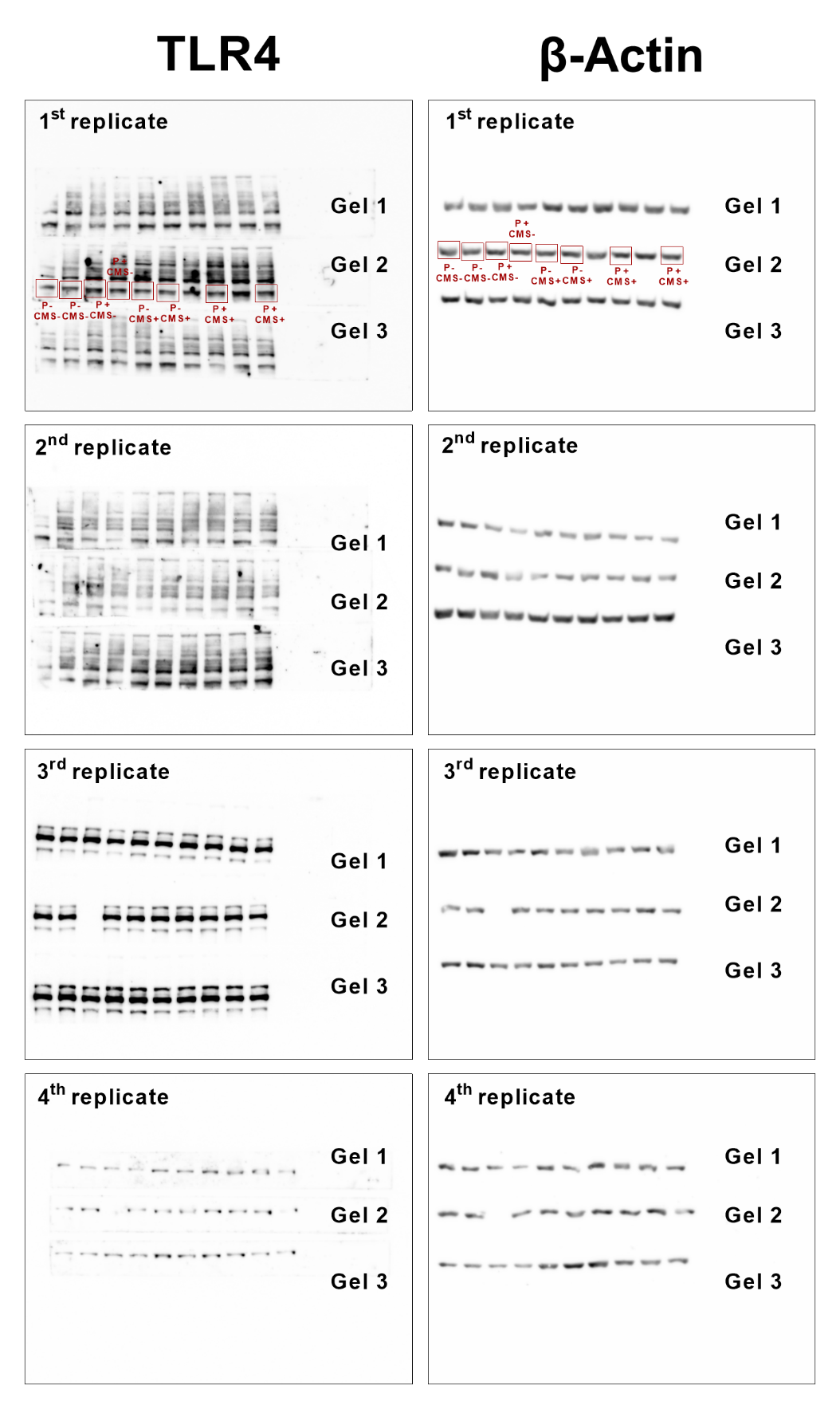


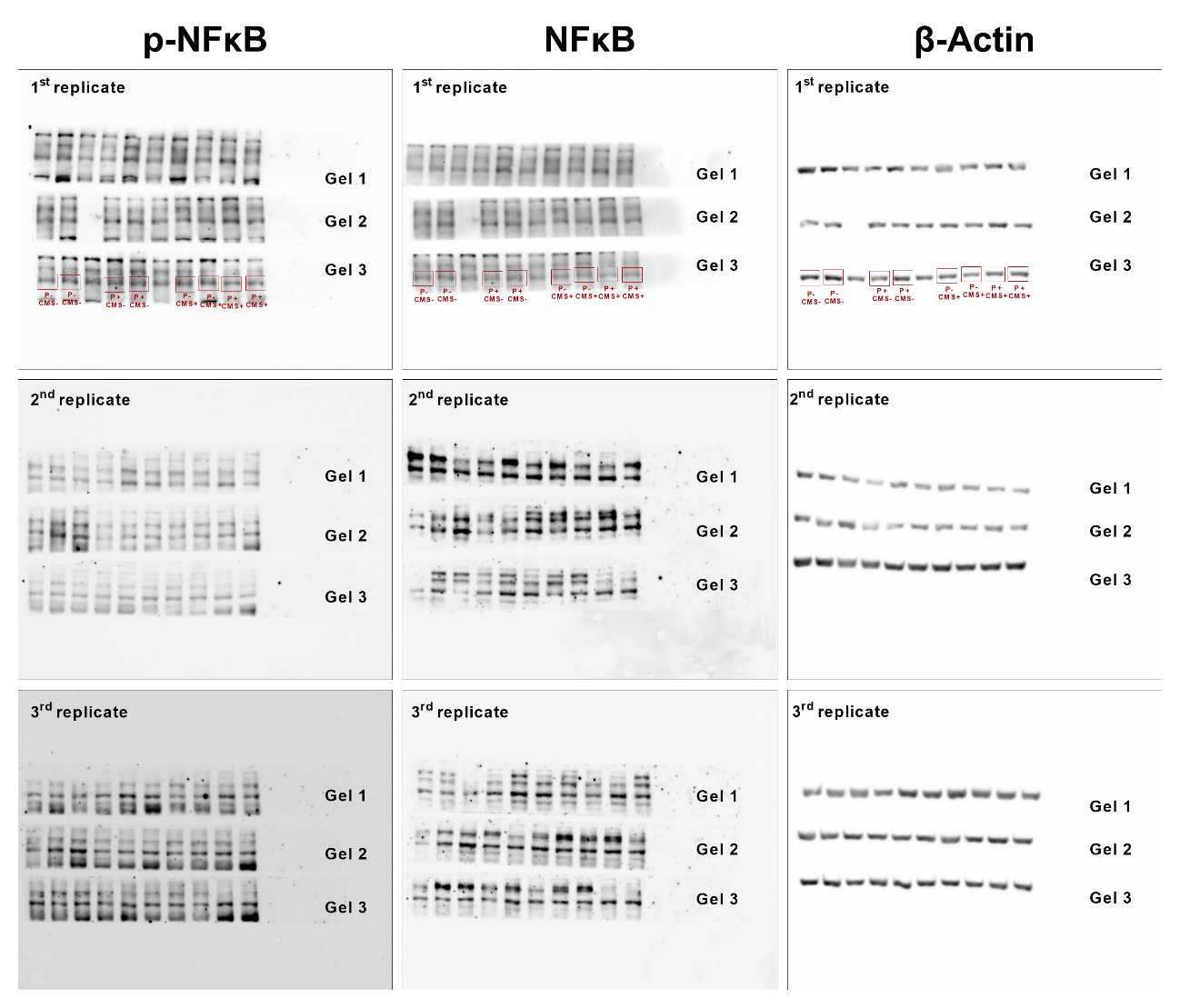


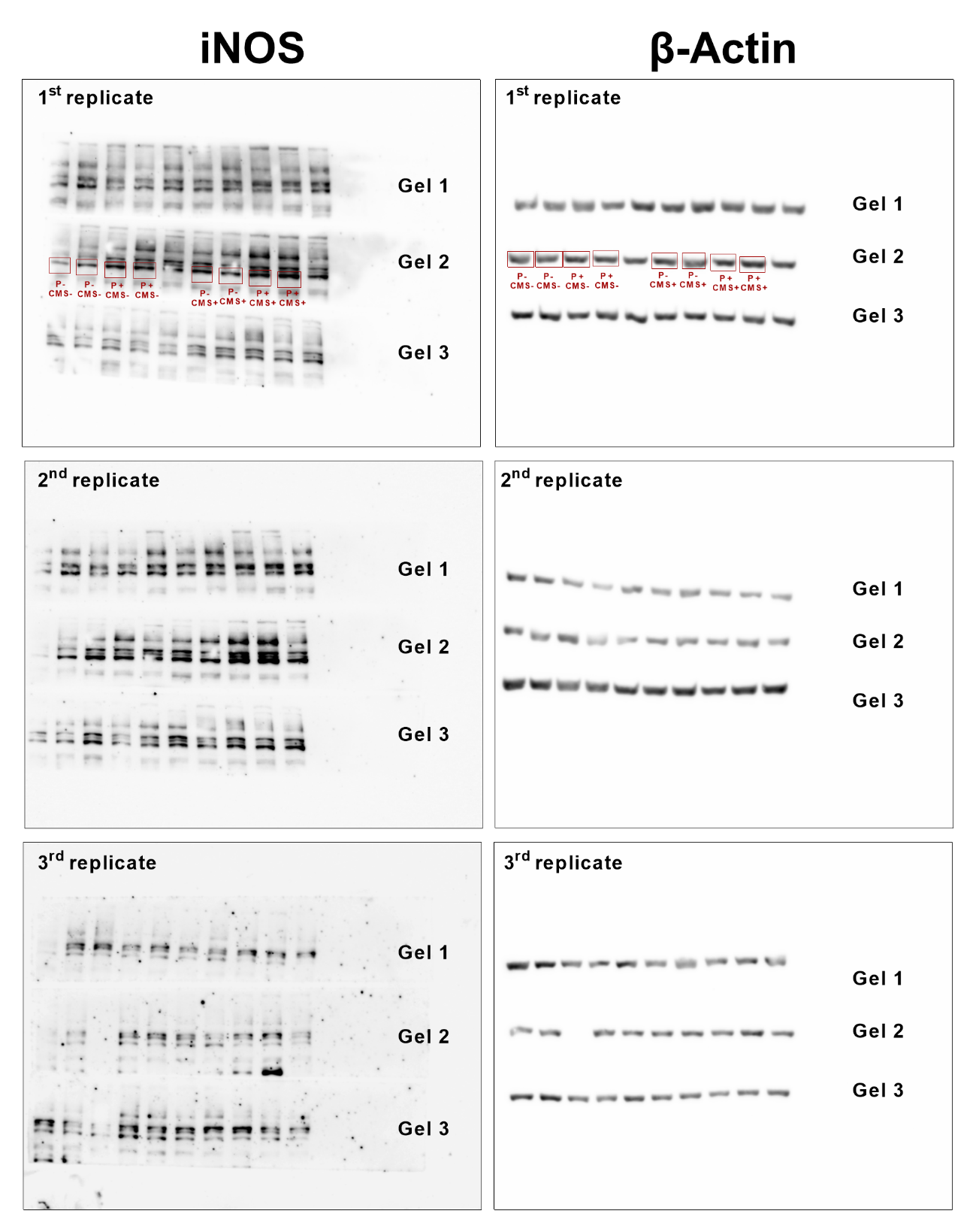


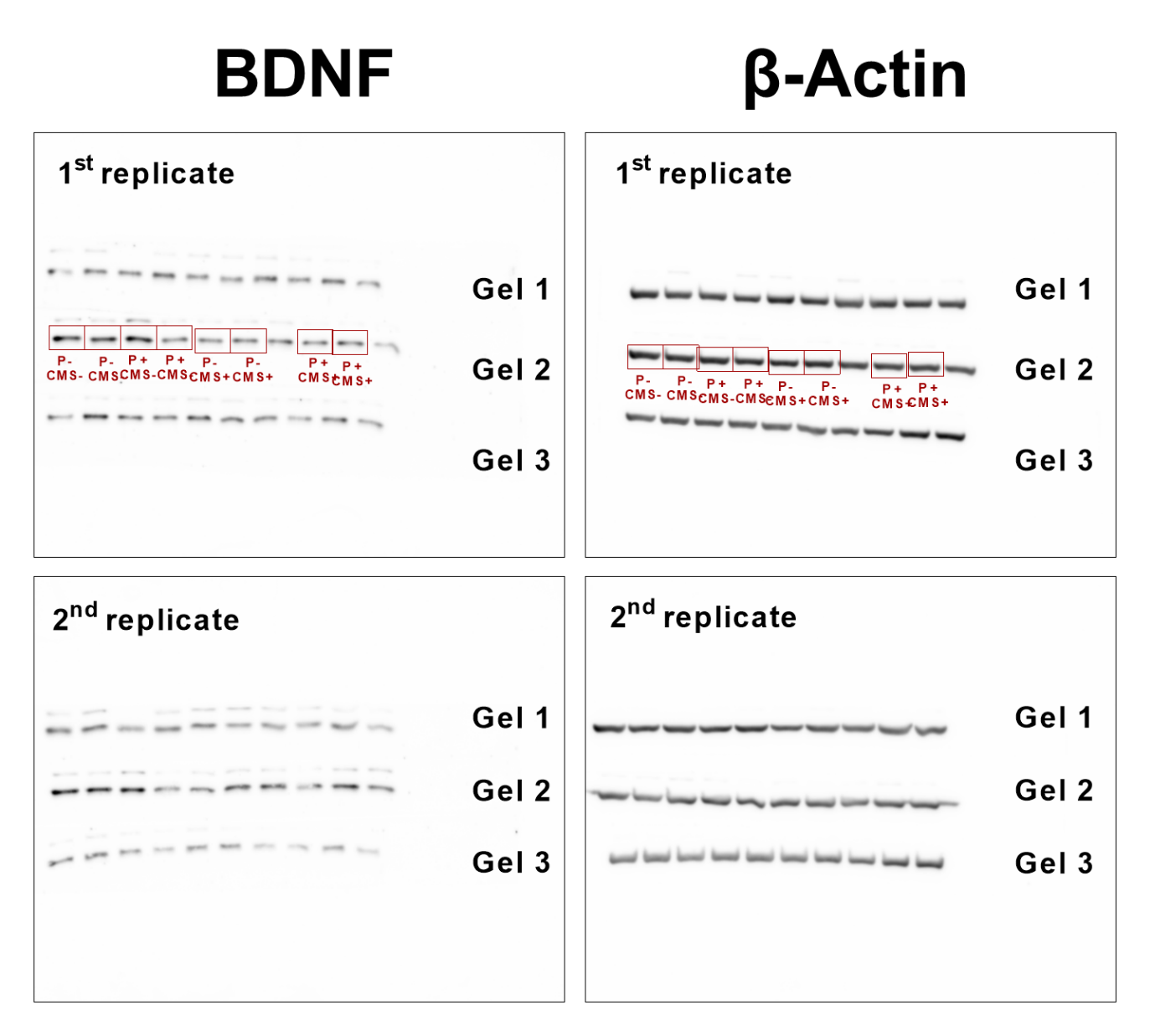


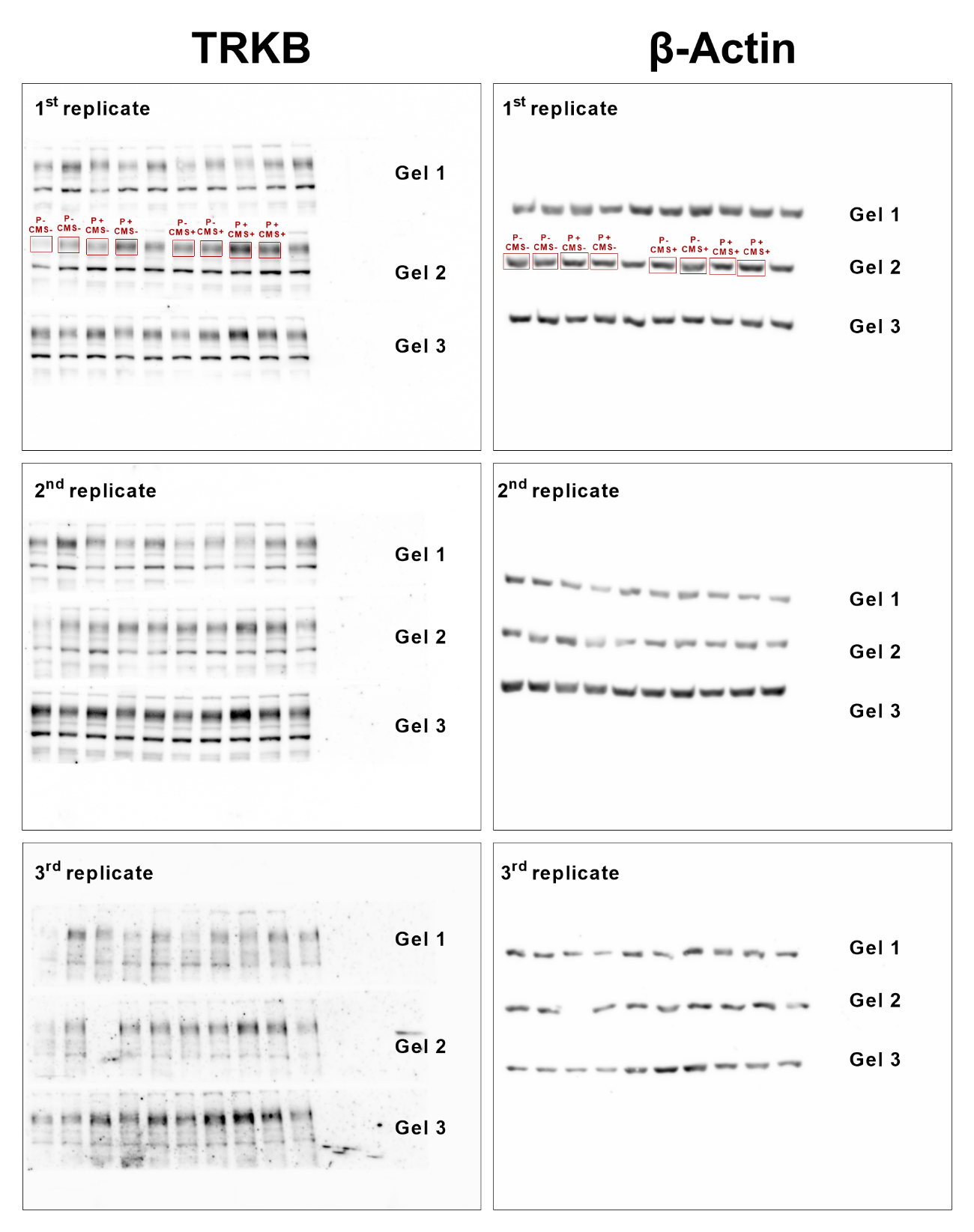


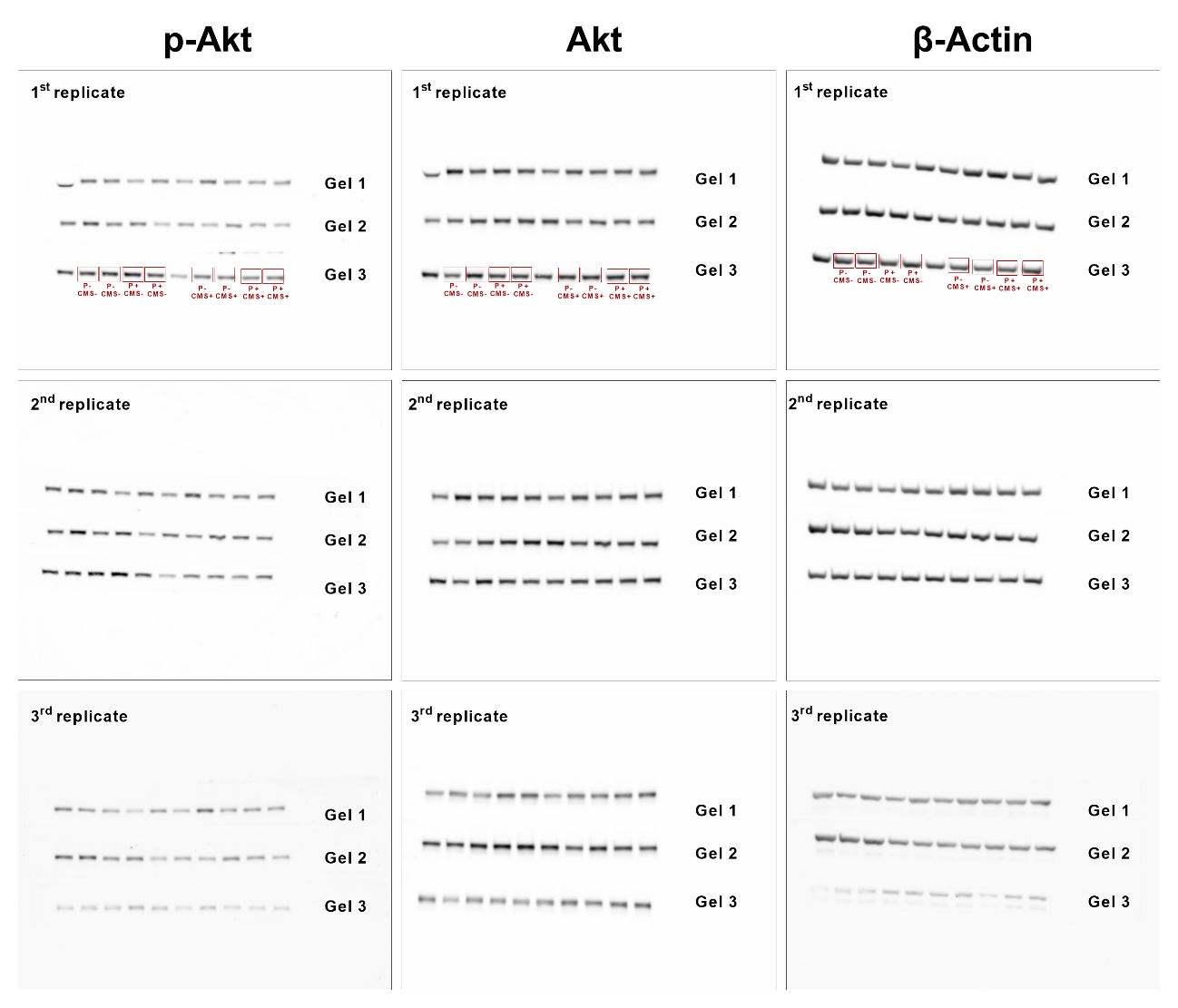


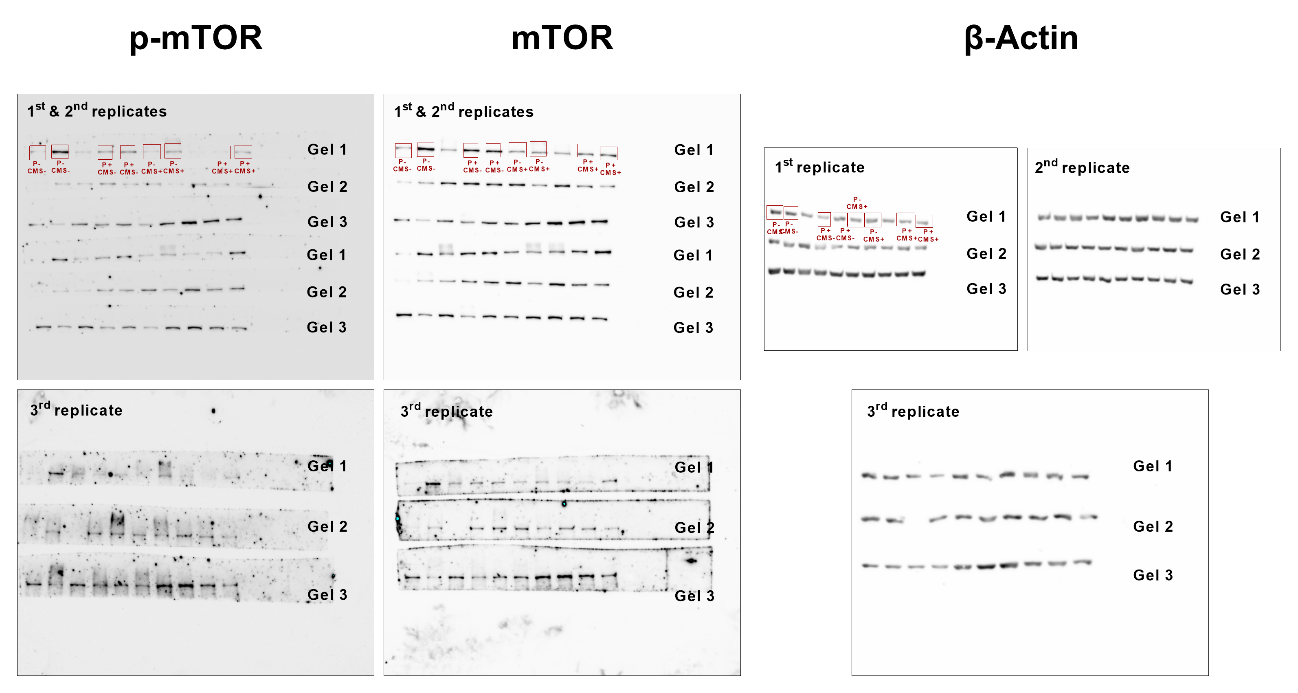


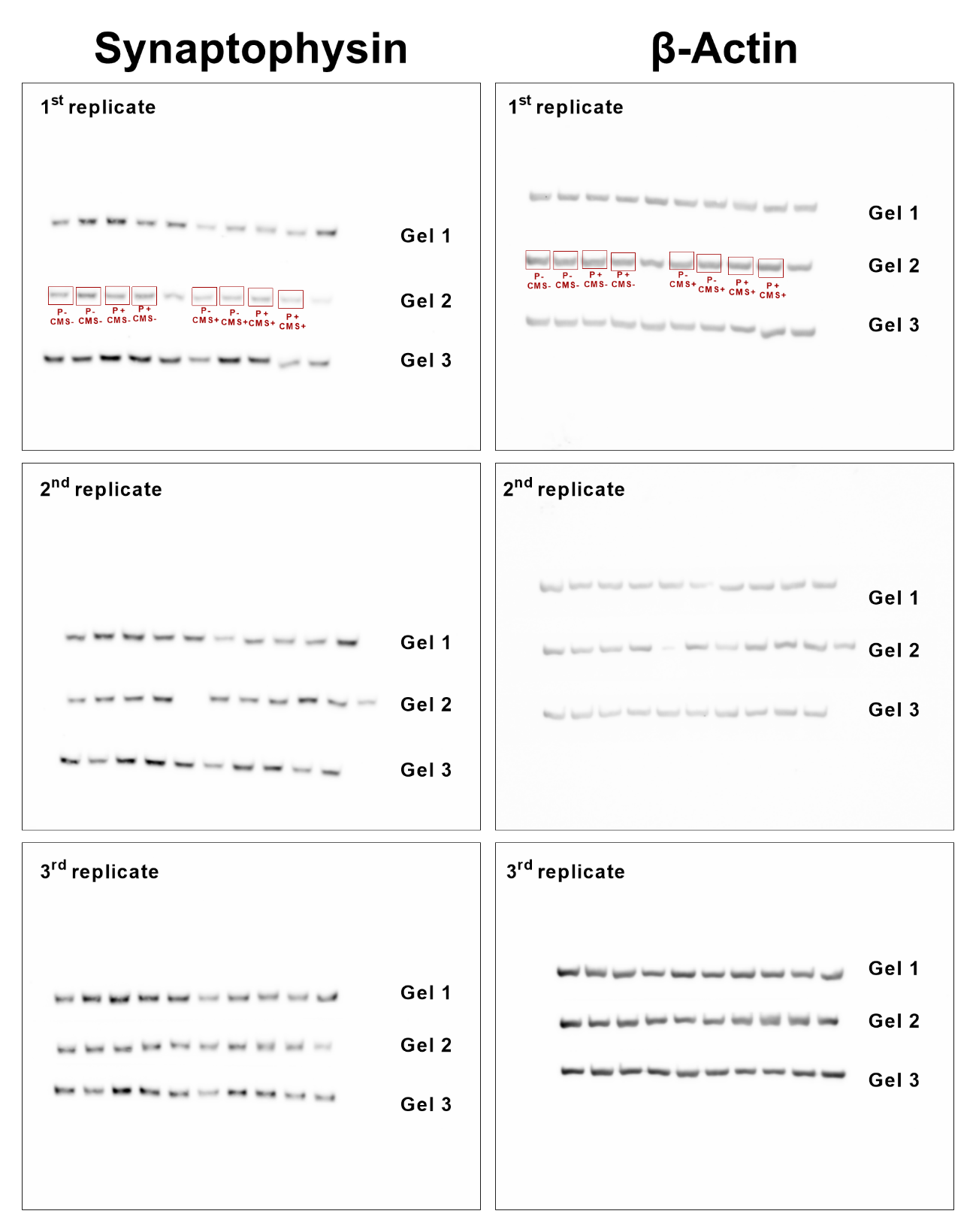


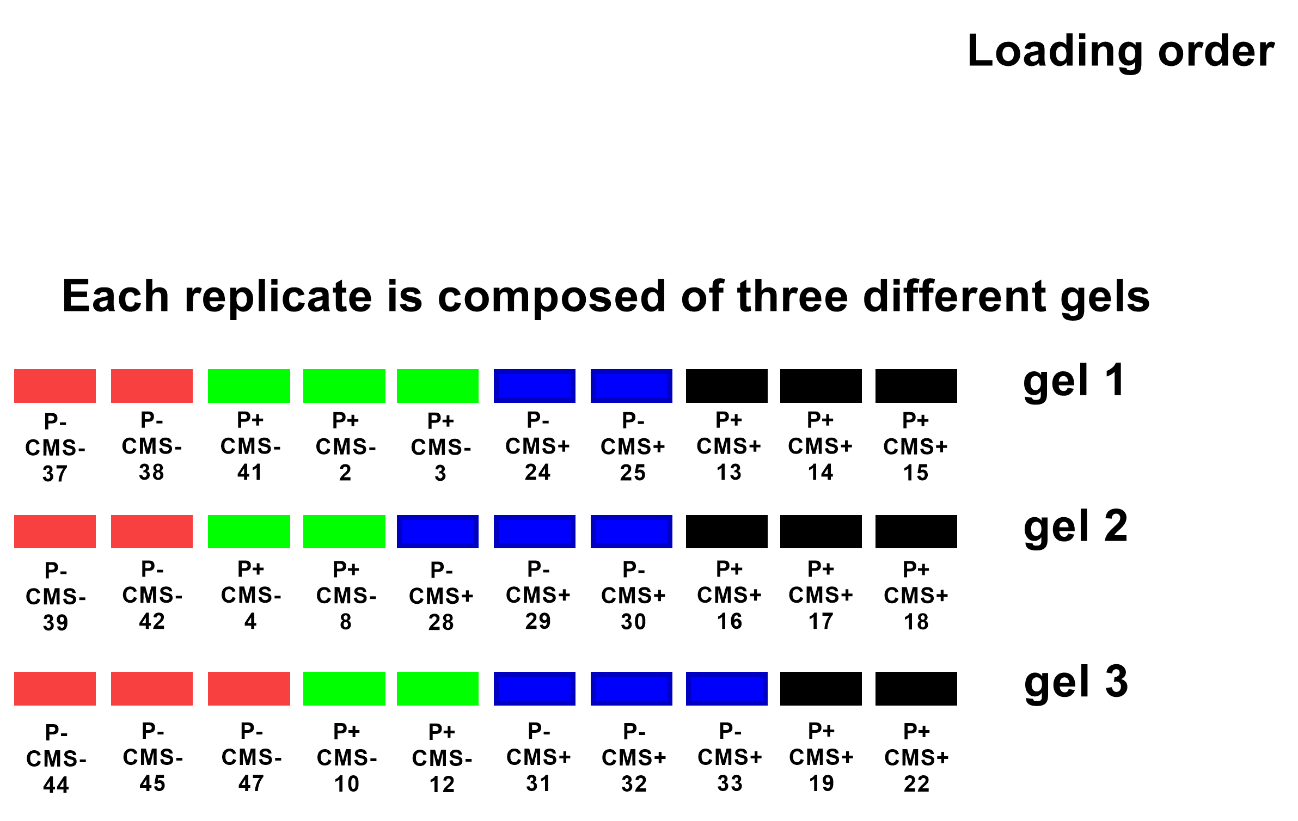

Supplement: Supplementary file 1 — Data S1: cns70669‐sup‐0001‐DataS1.docx. [file CNS-31-e70669-s001.docx]
